# A clinical in-hospital prognostic score for acute exacerbations of COPD

**DOI:** 10.1186/s12931-014-0099-9

**Published:** 2014-08-27

**Authors:** Nicolas Roche, Jean-Michel Chavaillon, Cyril Maurer, Mahmoud Zureik, Jacques Piquet

**Affiliations:** Respiratory and Intensive Care Medicine department, Cochin Hospital Group, APHP, University Paris Descartes (EA2511), 75005 Paris, France; Respiratory medicine and intensive care department, Antibes General Hospital, 06606 Antibes-Juan les Pins, France; Respiratory medicine department, Le Raincy-Montfermeil hospital, 93370 Montfermeil, France; INSERM Unit 700, Xavier Bichat University, 75018 Paris, France

**Keywords:** Acute exacerbation, Prognosis, Score, Pulmonary Disease, Chronic Obstructive

## Abstract

**Background:**

The use of a severity score to help orientation decisions could improve the efficiency of care for acute exacerbations of COPD (AECOPD). We previously developed a score (‘2008 score’, based on age, dyspnea grade at steady state and number of clinical signs of severity) predicting in-hospital mortality in patients with AECOPD visiting emergency departments (EDs). External validity of this score remained to be assessed.

**Objectives:**

To test the predictive properties of the ‘2008 score’ in a population of patients hospitalized in medical respiratory wards for AECOPD, and determine whether a new score specifically derived from this population would differ from the previous score in terms of components or predictive performance.

**Methods:**

Data from a cohort study in 1824 patients hospitalized in a medical ward for an AECOPD were analyzed. Patients were categorized using the 2008 score and its predictive characteristics for in-hospital mortality rates were assessed. A new score was developed using multivariate logistic regression modeling in a randomly selected derivation population sample followed by testing in the remaining population (validation sample). Robustness of results was assessed by case-by-case validation.

**Results:**

The 2008 score was characterized by a c-statistic at 0.77, a sensitivity of 69% and a specificity of 76% for prediction of in-hospital mortality. The new score comprised the same variables plus major cardiac comorbidities and was characterized by a c-statistic of 0.78, a sensitivity of 77% and specificity of 66%.

**Conclusions:**

A score using simple clinical variables has robust properties for predicting the risk of in-hospital death in patients hospitalized for AECOPD. Adding cardiac comorbidities to the original score increased its sensitivity while decreasing its specificity.

**Electronic supplementary material:**

The online version of this article (doi:10.1186/s12931-014-0099-9) contains supplementary material, which is available to authorized users.

## Introduction

Acute exacerbations of chronic obstructive pulmonary disease (AECOPD) are major events in the long-term course of the disease since their repetition is associated with impaired lung function, health status and survival and markedly increased health care costs [1]. On the short-term, they impair often notably health status and expose to risks of acute respiratory failure and death. Home-based care has been shown to represent a valuable alternative for many patients visiting emergency departments (EDs), allowing to avoid or shorten hospital stays [2]. However, most patients with AECOPD visiting EDs are hospitalized. In that context, assessing the severity of AECOPD is mandatory to guide decisions of orientation (home, hospital medical ward or intensive care unit -ICU-) as well as intensity of monitoring, treatment and follow-up during and after the acute episode [3]. Knowledge of all relevant prognostic factors is of major importance to determine the safest and most cost-effective setting for patient care. Several scores (e.g., the BAP-65 or DECAF scores) have been specifically developed to allow risk prediction in patients hospitalized for acute exacerbations of COPD, and were shown to perform better than scores primarily developed for other respiratory illnesses (i.e., community-acquired pneumonia) such as CURB-65 [4,5]. However, studies in this area are heterogeneous in terms of measured variables. In addition, some data (such as biomarkers) [6] might not be readily available in all settings, underlining the need for simple, purely clinical prediction rules. In other diseases such as community-acquired pneumonia, the use of scores (e.g., the Pneumonia Severity Index) was shown to improve the efficiency of care by allowing to decrease hospitalization rates without increasing the risk of poor outcome [7].

In 2003–2004, we conducted a prospective cohort study of 794 patients with AECOPD recruited in 104 French emergency departments [8]. Among these patients, only 10.5% were discharged home; in the remaining, the in-hospital mortality rate was 7.4%. Independent predictors of in-hospital death were age, clinical signs of severity and baseline dyspnea grade at steady state. From these observations, we derived and validated a prognostic score (Table 1) using two randomly selected halves of the cohort (the derivation and validation cohorts). For convenience, this score will be subsequently called the ‘2008 score’ after its year of publication. Its discriminative capacity proved to be satisfactory with a c-statistic (which is analogous to the area under the receiver operating characteristic -ROC- curve) at 0.82 in the validation cohort. Therefore, we aimed at assessing the validity of the 2008 score in another cohort that was primarily built to assess the long-term (4 years) prognosis and prognostic factors of patients hospitalized in medical wards for an AECOPD [9]. Considering the marked differences in the mode of recruitment (hospital wards vs EDs), study period, patients’ characteristics and mortality rate (see Table 2) between the two cohorts, we postulated that demonstrating a satisfactory discriminative capacity of the 2008 score in this new cohort would allow to generalize its use.

**Table 1:** **Calculation of the 2008 score**

**2008 score**	**Points**
**Age**	
● < 70 years	0
● > = 70 years	1
**MRC (baseline, steady state)**	
●0-1	0
●2-3	1
●4-5	2
Number of signs of severity* at entry	
●0	0
●1-2	2
●3 and more	3

*signs of severity: cyanosis, use of accessory inspiratory muscles, paradoxical abdominal movement, asterixis, neurological impairment, lower limb edema.

Total score ranges from 0 to 6. Tertiles of the original (2008) population corresponded to scores of 0–1, 2–3 and 4–6, respectively.

**Table 2:** **Main differences between the population in which the 2008 score was developed and the present population. All p ≤ 0.001**

**Variable**	**Present population**	**2008 population**
**Sex, % women**	23.2	30.0
**Age** (years, mean ± SD )	70.3 ± 11.3	72.5 ± 11.8
**Smoking status, %**		
●Non smoker	6.6	19.3
●Ex smoker	60.8	49.9
●Smoker	32.6	30.8
**MRC dyspnea grade at steady state, %**		
●0/1	17.1	22.5
●2-3	69.9	46.2
●4	13.0	31.3
**Maintenance treatment, %**		
●LTOT	37.7	23.2
●Inhaled corticosteroids	72.4	43.8
●Oral corticosteroids	7.8	13.8
●Bronchodilators	86.6	69.6
**Clinical signs of severity, %**		
●Cyanosis	22.1	29.0
●Use of accessory inspiratory muscles	27.9	50.9
●Paradoxical abdominal movement	8.6	36.8
●Asterixis	1.8	7.8
●Neurological impairment	6.9	11.8
●Lower limb edema	13.3	23.3
**Number of clinical signs of severity, %**		
●None	44.1	27.6
●1 -2	45.9	47.7
● ≥ 3	10.0	24.7
**Mortality (%)**	2.5	7.9

We also questioned whether a new score developed using data from patients of the new cohort would be different and more accurate to predict prognosis than the 2008 score. Thus, we applied the same methodology as in the previous study to develop a new score before comparing its components and performance to that of the 2008 score.

## Material and methods

### Study design

Details on study design and collected data have been published previously [9,10]. From October 2006 to June 2007, lung specialists from 68 French general hospitals consecutively included 1,824 patients newly admitted in their respiratory medicine department for an acute exacerbation of COPD, regardless of the source of admission (i.e., direct referral or via emergency department, intensive care unit [ICU], outpatient clinic, or another department or hospital). To be selected patients had to satisfy four conditions: (1) having or being strongly suspected of having COPD; for the second category, i.e. those suspected of having COPD, the diagnosis had to be confirmed subsequently by senior lung specialists during hospital stay; (2) presenting with an acute increase in at least one cardinal respiratory symptom (cough, sputum production or purulence, dyspnea) that was not immediately attributable to an alternative diagnosis such as pulmonary edema, pulmonary embolism, etc.; and (3) presenting with a condition considered sufficiently worrying in terms of duration and intensity to warrant hospitalization. All patients were informed of the objectives and requirements of the study and gave their oral consent before inclusion, as required by French regulation on observational studies. The protocol was approved by the Ethics Committee of the French-Language Society of Pulmonology.

### Collected data

Collected data addressed the characteristics of (i) the patient (i.e., anthropometric and sociodemographic characteristics, medical history, habits); (ii) underlying COPD (clinical characteristics, FEV_1_ and management before the exacerbation); (iii) the acute exacerbation (etiology and clinical characteristics on admission); (iv) hospital care (admission modalities, care pathways, treatments of the acute exacerbation) and (v) outcomes (duration of hospital stay, vital status at discharge). Spirometry results used in the analyses were the most recent prior to the exacerbation. Collected comorbidities of interest were ischemic heart disease, left heart failure, pulmonary hypertension, gastro-esophageal reflux, lung cancer, associated respiratory diagnoses (asthma, bronchiectasis, sleep apnea syndrome, obesity-hypoventilation syndrome).

### Statistical analyses

Standard SAS® procedures (SAS Institute, Cary, NC, USA) were used for statistical analysis. A description of the population was performed for all variables. Characteristics of this population were compared to that of the 2008 population using Chi^2^-test for categorical variables and Student t test or analysis of variance (ANOVA) (normal distribution) or non-parametric tests (non-normal distribution) for quantitative variables. The same methods were used to analyze factors associated with in-hospital death in the present population.

Two approaches were then used to test the validity of the 2008 score: the first was its implementation in the present population, allowing to calculate mortality rates by score category and to assess its predictive properties; the second was the development of a new score based on the characteristics of the present population, to assess whether this new score would be different from the 2008 score in terms of components and predictive performance.

### Application of the 2008 score in the studied population

To assess the global predictive capacity of the 2008 score in the present population, the score was calculated for each patient and the population was divided into three groups based on the scores observed in tertiles of the 2008 population (i.e., 0–1 for group 1, 2–3 for group 2 and 4 or more for group 3). Mortality rates were compared between groups, and sensitivity and specificity of the score for prediction of mortality was assessed. The c-statistic, which is analogous to the area under the receiver operating characteristic (ROC) curve, was then calculated.

### Development of a new score

To develop the new score, we used data collected during the first 24 hours of hospitalization in the studied population (see above). Two methods were used: the first (described below) was based on randomly selected derivation and validation cohorts, as for the development of the 2008 score, while the second (presented in the online Additional file 1) used score derivation and case-by-case validation in the whole population.

To implement the first method, the population was randomly split into two groups: a derivation cohort (n = 912) and a validation cohort (n = 912). In the derivation cohort, a backward stepwise logistic regression procedure was used, in which variables that were significant at p < 0.25 in univariate analyses were introduced. This p-value threshold was chosen to ensure without any doubt that no relevant variable would be missed: indeed, some variables that were thought to be of potential clinical importance such as coexisting asthma or sleep apnea syndrome were associated with mortality with a p value of about 0.20 in univariate analysis. Variables were eligible for inclusion in the final model if they were significantly associated with death at a two-tailed p-value of less than 0.05. As previously described [11–13], the multivariate model then allowed developing a point-based risk scoring system: the number of points assigned to each risk factor was obtained by dividing each Beta coefficient by the smallest Beta coefficient significantly different from 0 and rounding to the nearest integer. A risk score was assigned to each participant in both derivation and validation cohorts by summing the number of points corresponding to each risk factor. Model discrimination was assessed by the c statistic. In both derivation and validation cohorts, subjects were divided into three groups corresponding to tertiles of the score in the whole population, and mortality rates were compared between groups.

## Results

### Patients and in-hospital outcomes

Recruited patients reported recent acute and sustained onset or increase in dyspnea in 95% of cases, cough in 65% and sputum volume and/or purulence in 55%. Table 2 shows the main characteristics of the 1,824 patients at entry, compared to the 2008 population. Age was <60 years in 19.9% and ≥80 years in 23.2%.There was no difference in cumulative smoking (43.5 ± 25.2 vs 46.4 ± 30.0 pack-years), percentage of patients complaining of an increase in dyspnea (95.2% vs 99.2%) or of purulent sputum (42.6% in each population), percentage of patients in whom COPD had been previously diagnosed (84.1% vs 84.6%). Only 6.6% of patients were never smokers, 32.6% were still active smokers. Lung function data were obtained for 1615 patients (88.9%), with a delay of 17.4 ± 23.9 months between spirometry and hospital admission for AECOPD. FEV_1_ was 45.6 ± 17.7 L/s, with the following distribution of GOLD severity of airflow obstruction: GOLD I: 4.5%; GOLD II: 32.7%; GOLD III: 43.5%; GOLD IV: 19.4%. This could not be compared to the 2008 population, in which lung function data were not available in most cases in the context of the Emergency Department setting. Comorbidities, which were not recorded in details in the 2008 population either, were frequent in the present population: ischemic heart disease (IHD): 19.0%, left heart failure (LHF): 12.7%, systemic hypertension: 35.1%, asthma: 13%. BMI was ≤20 kg/m^2^ in 20.3% of patients and >25 kg/m^2^ in 45.1%.

In-hospital mortality rate was 2.46% (n = 45). Hospital length of stay was12.1 ± 9.7 days, i.e., similar to what was observed in the 2008 population (11.8 ± 14.1 days).

### Application of the 2008 score

Patients were distributed in three groups based on their 2008 score. These groups were defined based on the tertiles of the score in the original (2008) population. Mortality rates are shown in Table 3, and Table 4 presents the discriminative performance of the score, which was characterized by a c-statistic at 0.77, with a sensitivity of 69% and a specificity of 76%. In Tables 3 and 4, these results are compared to that obtained with the new score (see below).

**Table 3:** **Mortality rates by categories of the 2008 score and the new score**

**2008 score**			**New score**		
**Categories**	Groups*	**Deaths n (%)**	**Tertiles**	**Deaths n (%)**	
				**Derivation cohort**	**Validation cohort**
**0-1 point**	692 (37.9%)	5 (0.7%)	**0 point**	1 (0.3%)	0 (0%)
**2-3 points**	672 (36.8%)	11 (1.6%)	**1-2 points**	4 (1.3%)	5 (1.6%)
**4-6 points**	460 (25.2%)	29 (6.3%)	**3-9 points**	18 (5.8%)	17 (5.5%)

*defined with reference to the distribution (i.e., tertiles) of the 2008 score in the 2008 population.

The new score was built using data collected in the studied population.

**Table 4:** **Performance of the scores in the present population (95**% **CI)**

	**2008 score**		**New score**	
**c-statistic**	0.77	(0.72-0.81)	0.79	(0.74-0.82)
**Sensitivity**	0.64	(0.48-0.78)	0.77	(0.61-0.86)
**Specificity**	0.76	(0.74-0.78)	0.67	(0.65-0.69)

### Development of the new score

Variables associated with survival status at the end of hospital stay in univariate analyses performed in the whole studied population are presented in Additional file 1: Tables S1 and S2. Among these, multivariate analyses in the derivation cohort identified age, number of clinical signs of severity, mMRC dyspnea grade at steady state and presence of cardio-vascular comorbidities (ischemic heart disease and/or left heart failure) as being independently associated with mortality (Table 5). Table 6 shows how the new score was calculated based on these results. Mortality rates in the three tertiles of the score are shown in Table 3 for the derivation and validation cohorts, compared to those observed with the 2008 score in the three groups of the whole studied population defined with reference to distribution (i.e., tertiles) of this score in the 2008 population. The discriminative performance of the new score in the derivation and validation cohorts is presented in Table 4, compared to that of the 2008 score. The c-statistics were at 0.79 and 0.78 in the derivation and validation cohorts, respectively, with a sensitivity of 77% in both cohorts and specificities of 67% and 66%, respectively. Results of the case-by-case development and validation method were identical and are presented in Additional file 1: Table S3.

**Table 5:** **Results of multiple logistic regression identifying factors independently associated with mortality in the studied population**

	**Odds-Ratio**	**95% CI**	**P value**
Age (per year)	1,06	1,02-1,10	0,002
mMRC grade > 2 vs 0-1	3,91	1,70-8,96	0,001
Cardiovascular comorbidity (IHD and/of LHF)	2,28	1,06-4,91	0,036
Clinical signs of severity during the first 24 hours (per sign)*	1,34	1,18-1,53	<0,001

*IHD* : Ischemic heart disease; *LHF*: Left heart failure. *among the following: use of accessory inspiratory muscles, asterixis, neurological impairment, paradoxical abdominal movement, lower limb edema, cyanosis.

**Table 6:** **Calculation of the new score, based on results of multivariate analysis of factors associated with in-hospital death in the studied population**

**New score**	**Points**
**Age**	
● < 60 years	0
●60-80 years	1
●80 years	2
**MRC (baseline, steady state)**	
●0-2	0
● > 2	3
**Cardiovascular disease**	
●No	0
●Yes	2
**Number of signs of severity (first 24 hours)**	
●0	0
●1-2	1
●3 and more	2

Range of the total score: 0–9.

## Discussion

The initial score published in 2008 appears to have a good overall performance in the population studied here, as assessed by a similar discriminative performance (as assessed using the c-statistic) as in the original (2008) population. However, the 2008 score was less sensitive (but more specific) for prediction of mortality than a new score specifically derived from data of the studied population and additionally integrating cardiac comorbidities (which were not consistently recorded in the original −2008- population). Therefore, since the main purpose of such score should be to identify as much at-risk patients as possible, the new version might be preferable, at least for a population of patients hospitalized in a medical ward for an acute COPD exacerbation.

### Strengths and limitations, applicability of results

One important strength of this study is that collected data were those usually available immediately at presentation in patients with exacerbations of COPD, to ensure that the study conditions reflected what happens in the real-life. Actually, FEV_1_ or arterial blood gas tensions did not add anything to the purely clinical variables that constituted both the 2008 score and the new score. We did not include any biomarker in the assessment of studied patients, since these variables might not be available in all settings yet nor immediately at patients’ entry, which could limit the generalizability of a score relying on them. Indeed, variables of interest were collected rather exhaustively; however, this would have been quite different outside of a clinical survey (see below). Most scores developed in patients with AECOPD actually rely on some biological variables [4,5]. Even if these variables are available in most settings, this may increase the delay between arrival in the ED and risk stratification and, therefore, decisions regarding patients’ orientation. Another strength is that two methods of development and validation of the new score were used, with identical results. This is reassuring as to the robustness of this score.

Some limitations also have to be considered when interpreting the results. Firstly, this study was performed in patients admitted to a medical respiratory ward for AECOPD, whatever their care pathway had been before. Therefore, although these patients were otherwise unselected, they are not representative of patients visiting Emergency Departments of admitted in Intensive Care Units or in non-specialized (e.g., internal medicine) wards. Indeed, the main purpose of these analyses was not to build a new score to be applied in a specific population, but to determine whether a score originally developed in patients visiting EDs could be applied in other settings with satisfactory properties. Secondly, patients recruited in a medical ward are by nature those who have survived the first step of care for their exacerbation, e.g. the ED care. Indeed, in our 2008 study, 10 patients died in the ED (1.25%) and 7 (0.9%) died in the ICU in which they had been transferred from the ED [9]. Overall, 28,8% of all in-hospital deaths observed in that study occurred before the patient could reach the medical ward, and would therefore not have been captured in the present study.

### Variables included in the 2008 score and the new score

Besides differences in the weighting of some variables, the main difference between the two scores was that the new one accounted for cardio-vascular major comorbidities, i.e., IHD and LHF. This is likely explained by the lack of details on cardio-vascular comorbidities in the 2008 population, obviously preventing them from being reliably included in the score calculation. All other contributing variables (i.e., age, baseline mMRC grade at steady state and number of clinical signs of severity at entry) were the same, which confirms the strength of their association with mortality. This underlines the major importance of recording them at entry, which is not always done. Indeed, an audit in UK hospitals found marked heterogeneity of variables recorded in patients hospitalized for AECOPDs [14]. The European Respiratory Society audit on AECOPDs also found noticeable discrepancies between settings and countries in the way AECOPDs are assessed and cared for [15]. Whether this translates in differences in outcomes or cost-effectiveness of care remains to be demonstrated.

Interestingly, the importance of cardio-vascular comorbidities as prognostic factors in AECOPD is in line with the high proportion of cardiovascular deaths in patients with COPD [16,17]. Indeed, cardiovascular comorbidities are frequent in these patients [18,19], due to common risk factors (smoking, age) and maybe also to specific pathophysiological mechanisms involving COPD-associated systemic inflammation [20–22]. COPD is also known to impair the prognosis of cardiovascular diseases such as IHD or CHF [23,24] while a low FEV1 increases the risk of atheroma [25] and cardiovascular death [26]. In addition, ischemic cardiovascular events are frequent during and early after AECOPD [27,28].

### Is the use of a score able to improve outcomes of AECOPD?

In the field of AECOPD outside ICUs, it has never been demonstrated that using such a score has an effect on the appropriateness of medical decisions. In other acute respiratory diseases such as community acquired pneumonia (CAP), the use of scores (e.g. the Pneumonia Severity Index) was shown to improve the efficiency of patients orientation through a decrease in hospitalizations without any increase in the risk of poor outcome [7]. However, it might be argued that AECOPD and CAP are conceptually very different diseases: CAP are by definition of infectious origin while only about 50% of AECOPD are associated with identification of a pathogen [1]. In AECOPD, the main phenomenon is not parenchymal infection but airways obstruction. In addition, in AECOPD other etiologies of respiratory failure such as pulmonary embolism are frequent and frequently undiagnosed [29]. Thus, it could be hypothesized that building a simple clinical score would be more difficult in AECOPD, due to their marked heterogeneity. However, the scores we developed are not more complex than, e.g., the CRB-65. Conversely, in a recent study, Steer et al. developed a score called the DECAF score, relying on extended MRC Dyspnoea Score, eosinopenia, consolidation, acidaemia, and atrial fibrillation [5]. Using internal bootstrap validation, the score predicted mortality quite reliably, with an area under the receiver operator characteristic (ROC) curve of 0.86. In addition, the DECAF score was a significantly stronger predictor of mortality than CURB-65. However, this score relies not only on clinical features but also on some radiological and biological characteristics, which might limit its applicability for use at entry in the ED. Besides the variables included in these scores, several other clinical, biological or radiological data have been found to be of prognostic significance in AECOPD (regarding, e.g., the risks of death, need for mechanical ventilation or prolonged hospitalization) [3]. Adding biological or imaging variables might improve the predictive capacity of the scoring systems but, as mentioned above, this would compromise their use in the community setting and, even in the hospital, awaiting their results could delay the score-based decisions.

Interestingly, although the DECAF score comprises three non-clinical (i.e., biological or radiological) variables, the area under the ROC curve (0.86) in its development study was not considerably superior to that of our purely clinical score (0.79). Of note, the c-statistic of the BAP-65 score (which uses one biological variable, BUN) in the validation study by Shorr et al. was also 0.79 [30].

## Conclusions

In this study, we confirm in hospitalized patients the overall good predictive properties of a purely clinical score initially developed in patients visiting the ED for an AECOPD. Adding major cardiac comorbidities (IHD, LHF) to scoring items increased the sensitivity of the score for mortality prediction. Additional studies are required to determine whether the use of such scores increases the efficiency of care for AECOPD, both in hospital or community settings.

## Additional file

Additional file 1:
**Supplementary methods and tables: case by case score development and univariate analyzes.**

## Abbreviations

ANOVA: Analysis of variance
COPD: Chronic obstructive pulmonary disease
CPHG: French College of General Hospital Respiratory Physicians
FEV1: Forced expiratory volume in one second
GOLD: Global Initiative for chronic Obstructive Lung Disease
ICU: Intensive care unit
MRC: Medical Research Council
OR: Odds ratio
