# Supplementary material for: A clinical in-hospital prognostic score for acute exacerbations of COPD
Source: Respir Res. 2014 Aug 27;15(1):99. doi: 10.1186/s12931-014-0099-9 (PMC4244057; doi:10.1186/s12931-014-0099-9)
Supplement: Additional file 1: — Supplementary methods and tables: case by case score development and univariate analyzes. [file 12931_2014_99_MOESM1_ESM.docx]

**A clinical in-hospital prognostic score for acute exacerbations of COPD**

Nicolas ROCHE, MD, PhD (1), Jean-Michel CHAVAILLON, MD (2), Cyril MAURER, MD (3), Mahmoud ZUREIK, MD (4), PhD, Jacques PIQUET, MD (3)

(1) Respiratory and Intensive Care Medicine department, Cochin Hospital Group, APHP, University Paris Descartes, 75005 Paris, France; (2) Respiratory medicine and intensive care department, Antibes General Hospital, 06606 Antibes-Juan les Pins, France; (3) Respiratory medicine department, Le Raincy-Montfermeil hospital, 93370 Montfermeil, France; (4) INSERM Unit 700, Xavier Bichat University, 75018 Paris, France.

**Additional file 1: supplementary methods and tables: case by case score development and univariate analyzes**

**Supplement to the methods section: case by case development and validation of the new score.**

The whole population (n=1824) was used for the development of the second new score (case-by-case). A backward stepwise logistic regression procedure was used, in which variables that were significant at p<0.25 in univariate analyses were introduced. Variables were eligible for inclusion in the final model if they were significantly associated with death at a two-tailed p-value of less than 0.05. The multivariate model then allowed developing a point-based risk scoring system: the number of points assigned to each risk factor was obtained by dividing each Beta coefficient by the smallest Beta coefficient significantly different from 0 and rounding to the nearest integer. A risk score was assigned to each participant by summing the number of points corresponding to each risk factor. Model discrimination was assessed by the c statistic. Subjects were divided into three groups corresponding to tertiles of the score in the whole population, and mortality rates were compared between groups.

Of note, with this method the total correct classification derived from the dataset is somehow biased because individuals are used both to develop and calculate the performance of the index. One way of estimating the classification bias is to remove an individual from the data, re-estimate the model parameters, and then classify this individual based on the new estimated parameters. And so on, for all individuals in the data set. The bias estimation was thus calculated by the difference between the correct classification derived from the set of data and the correct classification obtained after removing each of all individuals. The results of this approach were very close to those obtained with the first approach (supplementary tables 3 and 4)

Supplementary table 1: factors associated with survival status at the end of hospital stay in the present population: univariate analyses for baseline variables.

| **Variable** |  | Alive |  |  | Dead | P value |
| --- | --- | --- | --- | --- | --- | --- |
|  | N | mean±SD or % |  | N | mean±SD or % |  |
| **Age (years)** | 1772 | **70.1± 11.2** |  | 45 | **77.6± 9.7** | <0.0001 |
| **Age (years)**, % | 1772 |  |  | 45 |  | <0.0001 |
| - <60 |  | **20.3** |  |  | **6.7** |  |
| - 60-79 |  | **57.2** |  |  | **42.2** |  |
| - ≥80 |  | **22.5** |  |  | **51.1** |  |
| Smoking status, % | 1772 |  |  | 45 |  | 0.026 |
| - Non-smoker |  | **6.7** |  |  | **4.4** |  |
| - Ex-smoker |  | **60.3** |  |  | **80.0** |  |
| - Smoker |  | **33.3** |  |  | **15.6** |  |
| Pack-years | 1605 | **43.4± 25.2** |  | 41 | **49.7± 25.5** | 0.11 |
| **Comorbidities, %** | 1772 |  |  | 45 |  |  |
| - Asthma |  | **13.3** |  |  | **6.7** | 0.20 |
| - Bronchiectasis |  | **6.8** |  |  | **13.3** | 0.09 |
| - Ischemic heart disease |  | **18.6** |  |  | **33.3** | 0.013 |
| - Left heart failure |  | **12.3** |  |  | **26.7** | 0.004 |
| - Secondary pulmonary hypertension |  | **7.1** |  |  | **17.8** | 0.007 |
| - Right heart failure |  | **4.2** |  |  | **17.8** | <0.0001 |
| - Sleep apnea syndrome |  | **7.4** |  |  | **2.2** | 0.19 |
| - Obesity-hypoventilation syndrome |  | **5.4** |  |  | **-** | 0.11 |
| - Lung cancer |  | **2.8** |  |  | **6.7** | 0.12 |
| **Baseline mMRC grade at steady state,%** | 1749 |  |  | 42 |  | <0.0001 |
| - 0/1 |  | **17.3** |  |  | **11.9** |  |
| - 2 |  | **36.2** |  |  | **4.8** |  |
| - 3 |  | **34.2** |  |  | **40.5** |  |
| - 4 |  | **12.2** |  |  | **42.9** |  |
| **Baseline lung function and gas exchange at steady state** |  |  |  |  |  |  |
| FEV1 % predicted | 1573 | **45.9± 17.7** |  | 39 | **36.3± 12.4** | 0.0009 |
| FEV1 % predicted, (%) | 1573 |  |  | 39 |  | 0.021 |
| - ≥80% |  | **4.5** |  |  | **2.6** |  |
| - [50%-80%[ |  | **33.2** |  |  | **12.8** |  |
| - [30%-50%[ |  | **43.2** |  |  | **51.3** |  |
| - ≤30% |  | **19.1** |  |  | **33.3** |  |
| PaCO2 ≥45 mmHg, % | 1393 | **34.7** |  | 38 | **50.0** | 0.05 |
| PaO2 <60 mmHg, % | 1401 | **25.0** |  | 38 | **42.1** | 0.017 |
| Long-term treatments, % |  |  |  |  |  |  |
| **OLD** | 1485 | **36.8** |  | 42 | **66.7** | <0.0001 |
| Tracheotomy | 1485 | **0.4** |  | 42 | **2.4** | 0.06 |
| History of invasive ventilation | 1485 | **0.5** |  | 42 | **2.4** | 0.09 |
| Oral corticosteroids | 1485 | **7.3** |  | 42 | **23.8** | <0.0001 |
| **Delay since diagnosis of COPD** | 1428 | **102.2± 88.4** |  | 41 | **121.0± 83.5** | 0.18 |
| GOLD severity of airflow obstruction, % | 1365 |  |  | 34 |  | 0.038 |
| - I |  | **2.6** |  |  | **2.9** |  |
| - II |  | **30.5** |  |  | **11.8** |  |
| - III |  | **45.7** |  |  | **47.1** |  |
| - IV |  | **21.1** |  |  | **38.2** |  |

Supplementary table 2: factors associated with survival status at the end of hospital stay in the present population: univariate analyses for the history of exacerbations and characteristics of the acute exacerbation.

| **Variable** |  | Alive |  |  | Dead | P value |
| --- | --- | --- | --- | --- | --- | --- |
|  | N | mean±SD or % |  | N | mean±SD or % |  |
| AECOPD during the last 12 months, % | 1461 |  |  | 42 |  | 0.006 |
| - 0-1 |  | **49.3** |  |  | **28.6** |  |
| - 2 or more |  | **50.7** |  |  | **71.4** |  |
| Hospitalization for AECOPD during the last 12 months, % | 1461 |  |  | 42 |  | 0.016 |
| - 0 |  | **42.3** |  |  | **16.7** |  |
| - 1 or more |  | **51.7** |  |  | **83.3** |  |
| Etiology of the AECOPD, % | 1771 |  |  | 45 |  |  |
| - Left heart failure |  | **7.8** |  |  | **15.6** | 0.06 |
| - Pulmonary embolism |  | **0.4** |  |  | **2.2** | 0.07 |
| Symptoms of exacerbation, % |  |  |  |  |  |  |
| - Increased cough | 1771 | **65.8** |  | 45 | **37.8** | <0.0001 |
| Signs of severity, % | 1771 |  |  | 45 |  |  |
| - Dyspnea at rest |  | **75.6** |  |  | **88.9** | 0.040 |
| - Cyanosis |  | **21.9** |  |  | **33.3** | 0.07 |
| - SpO2 < 90% |  | **43.4** |  |  | **64.4** | 0.005 |
| - Use of accessory inspiratory muscles |  | **27.3** |  |  | **55.6** | <0.0001 |
| - Paradoxical abdominal motion |  | **8.4** |  |  | **17.8** | 0.026 |
| - Breathing frequency> 25/min |  | **33.5** |  |  | **53.3** | 0.005 |
| - Ineffective cough |  | **13.3** |  |  | **26.7** | 0.010 |
| - Heart rate > 110/min |  | **22.8** |  |  | **35.6** | 0.044 |
| - Arrhythmia |  | **3.5** |  |  | **13.3** | 0.0006 |
| - Hypotension |  | **2.3** |  |  | **11.1** | 0.0002 |
| - Lower limb edema |  | **13.1** |  |  | **24.4** | 0.027 |
| - Agitation, confusion |  | **5.1** |  |  | **11.1** | 0.08 |
| - Coma |  | **4.2** |  |  | **15.6** | 0.0003 |
| - Asterixis |  | **1.8** |  |  | **4.4** | 0.18 |
| **Dyspnea grade at entry,%** | 1668 |  |  | 44 |  | 0.038 |
| - 1 |  | **2.9** |  |  | **-** |  |
| - 2 |  | **8.6** |  |  | **2.3** |  |
| - 3 |  | **32.1** |  |  | **20.5** |  |
| - 4 |  | **56.4** |  |  | **77.3** |  |

Supplementary table 3: results of the score developed using case by case validation in the whole population: mortality by tertile and discriminative property.

|  | **Mortality** | | |
| --- | --- | --- | --- |
|  | **N** | **%** | |
| **Tertile 1 (0)** | 3 | 0.5 |  |
| **Tertile 2 (1-2 points)** | 8 | 1.3 |  |
| **Tertile 3 (3-9 points)** | 34 | 5.5 |  |
| **Discriminative performance for prediction of mortality** | | **95% CI** |  |
| **c-statistic** | 0.78 | 0.74-0.82 |  |
| **Sensitivity** | 0.76 | 0.61-0.86 |  |
| **Specificity** | 0.67 | 0.65-0.69 |  |

Supplementary table 4: the new score as obtained by case-by-case

| **Variable** | **Score** |
| --- | --- |
| Age |  |
| - <60 years | 0 |
| - 60<=Age>=80 years | 1 |
| - >80 years | 2 |
| MRC (baseline) |  |
| - 0-2 | 0 |
| - >2 | 3 |
| Cardiovascular comorbidity |  |
| - No | 0 |
| - Yes | 2 |
| Clinical signs of severity during the first 24 hours |  |
| • None | 0 |
| • 1-2 signs | 1 |
| • 3 signs or more | 2 |

Supplementary table 5: discriminative properties of the new score developed using the case-by-case method

|  | **Mortality rate (%)** | **95% CI** | |
| --- | --- | --- | --- |
| **Tertile 1 (0)** | 3 (0.5%) |  |  |
| **Tertile 2 (1-2 points)** | 8 (1.3%) |  |  |
| **Tertile 3 (3-9 points)** | 34 (5.5%) |  |  |
|  |  |  |  |
| **c-statistic** | 0.78 | 0.74-0.82 |  |
| - **Sensitivity** | 0.76 | 0.61-0.86 |  |
| - **Specificity** | 0.67 | 0.65-0.69 |  |
|  |  |  |  |
